# A Non-Synonymous *HMGA2* Variant Decreases Height in Shetland Ponies and Other Small Horses

**DOI:** 10.1371/journal.pone.0140749

**Published:** 2015-10-16

**Authors:** Mirjam Frischknecht, Vidhya Jagannathan, Philippe Plattet, Markus Neuditschko, Heidi Signer-Hasler, Iris Bachmann, Alicja Pacholewska, Cord Drögemüller, Elisabeth Dietschi, Christine Flury, Stefan Rieder, Tosso Leeb

**Affiliations:** 1 Institute of Genetics, Vetsuisse Faculty, University of Bern, 3001, Bern, Switzerland; 2 Agroscope, Swiss National Stud Farm, 1580, Avenches, Switzerland; 3 Swiss Competence Center of Animal Breeding and Genetics, University of Bern, Bern University of Applied Sciences HAFL & Agroscope, 3001, Bern, Switzerland; 4 Division of Experimental and Clinical Research, Vetsuisse Faculty, University of Bern, 3001, Bern, Switzerland; 5 Bern University of Applied Sciences, School of Agricultural, Forest and Food Sciences, 3052, Zollikofen, Switzerland; 6 Swiss Institute of Equine Medicine, Vetsuisse Faculty, University of Bern and Agroscope, 3001, Bern, Switzerland; University of Sydney, AUSTRALIA

## Abstract

The identification of quantitative trait loci (QTL) such as height and their underlying causative variants is still challenging and often requires large sample sizes. In humans hundreds of loci with small effects control the heritable portion of height variability. In domestic animals, typically only a few loci with comparatively large effects explain a major fraction of the heritability. We investigated height at withers in Shetland ponies and mapped a QTL to ECA 6 by genome-wide association (GWAS) using a small cohort of only 48 animals and the Illumina equine SNP70 BeadChip. Fine-mapping revealed a shared haplotype block of 793 kb in small Shetland ponies. The *HMGA2* gene, known to be associated with height in horses and many other species, was located in the associated haplotype. After closing a gap in the equine reference genome we identified a non-synonymous variant in the first exon of *HMGA2* in small Shetland ponies. The variant was predicted to affect the functionally important first AT-hook DNA binding domain of the HMGA2 protein (c.83G>A; p.G28E). We assessed the functional impact and found impaired DNA binding of a peptide with the mutant sequence in an electrophoretic mobility shift assay. This suggests that the HMGA2 variant also affects DNA binding *in vivo* and thus leads to reduced growth and a smaller stature in Shetland ponies. The identified HMGA2 variant also segregates in several other pony breeds but was not found in regular-sized horse breeds. We therefore conclude that we identified a quantitative trait nucleotide for height in horses.

## Introduction

Many biomedically or agriculturally important traits are quantitative. When studying the genetics underlying quantitative traits, their heritability and genetic architecture are important parameters [1]. Quantitative traits may be largely controlled by a few genes with large effects (“major genes”) as seen for instance in milk yield in cattle [2]. Other traits such as human body mass index [3] or height [4,5] are controlled by multiple up to hundreds of loci. The quantitative trait height has been studied intensively in humans and other species [5–8]. Height is often used as a model trait to develop methods for the discovery of quantitative trait loci (QTL). Although height has a very high heritability, it nonetheless has a complex architecture in humans. The most recent study, based on more than 250,000 people, identified 697 variants clustered in 423 loci associated with height [5]. These variants only explained as little as 20% of the heritable variance in humans.

Domestic animals usually display a different population structure than humans. They tend to be much more closely related and inbred and most breeds represent strictly isolated populations [9]. These isolated populations often have a relatively small effective population size and much less heterogeneity within breeds is observed compared to human populations. Height is a well-studied trait in many domestic animals [8,10–12]. Apart from being a model trait, height is also important in animal breeding programs. For instance in dogs or horses breed definitions include requirements for height and only individuals displaying the required height will be allowed for breeding. Modern horses (including ponies) range in height at withers from approximately 70–200 cm and thus represent an intraspecies phenotypic diversity that is only exceeded by the size variation in purebred dogs.

In dogs six variants explain about half of the size variation [8]. In horses four loci have been identified that explain about 83% of the height variance [7]. The loci identified in horses include the *NCAPG* locus on ECA 3, whose ortholog has also been found to be associated with height in cattle [12]. Further known height loci in horses are the *ZFAT* locus on ECA 9, *GH* on ECA11, and *HMGA2* on ECA 6. Another locus that is well-known to influence height in many species is the *IGF1* locus [13]. This locus has also been found in a selection signature study in the Franches-Montagnes (FM) horse breed and may represent a fifth height locus in horses [14].

Although several QTL for height in domestic animals have now been robustly validated, most of the underlying causative variants remain unknown. We report here a genome-wide association study for height in a small cohort of Shetland ponies that pointed to a role of the *HMGA2* locus. We studied the region in detail and identified a highly plausible candidate variant underlying this QTL.

## Results

### Height QTL mapping

**Fig 1:**
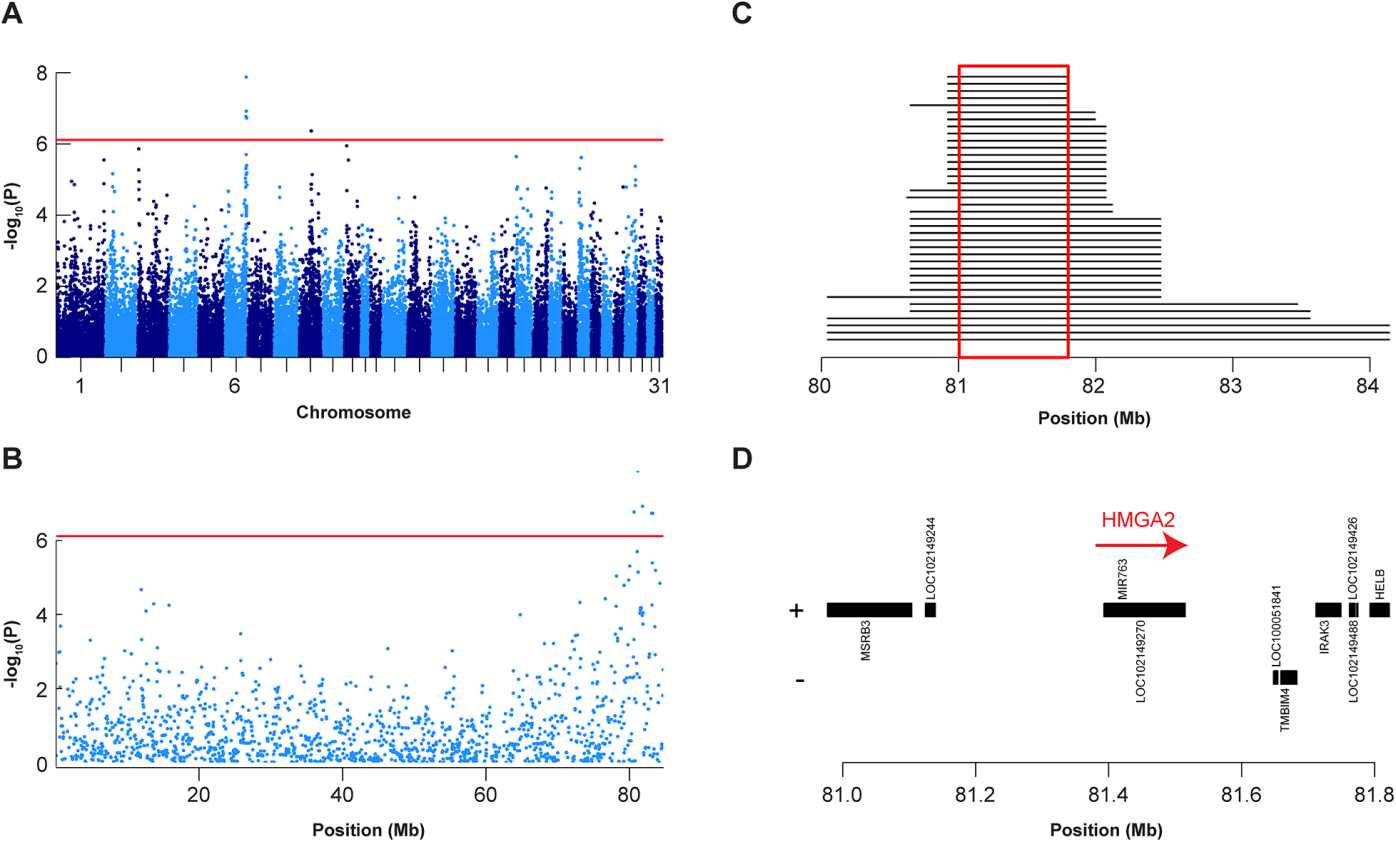
Mapping of height QTL in Shetland ponies. A: Manhattan plot of GWAS using 48 Shetland ponies and height at withers as a quantitative trait. B: Zoom of GWAS results on ECA 6. C: Haplotype analysis of the 19 smallest Shetland ponies. Each line represents one haplotype and the length of the lines represents the length of the shared haplotype block, which was defined based on a small Shetland pony that was homozygous for all significantly associated SNPs on ECA 6. D: NCBI MapViewer gene annotation in the critical interval (Annotation release 101, EquCab 2 genome assembly). The *HMGA2* gene is currently not annotated in the NCBI MapViewer. Its position and orientation was determined by comparisons with the orthologous human genome segment.

We performed a quantitative genome wide association study (GWAS) with respect to height at withers in Shetland ponies. We investigated 29 Shetland ponies with height at withers ≥87 cm and 19 Shetland ponies with height at withers <87 cm. Our final data set for the analysis thus consisted of 48 animals and their genotypes at 41,683 SNPs. This analysis revealed two QTL on ECA 6 and ECA 9 that exceeded the Bonferroni-corrected significance threshold (Fig 1, S1 Table). It has to be cautioned that the genomic inflation was high at 1.95 indicating substantial population stratification in our cohort. We also performed the initial association study with a mixed model analysis that corrects for the population stratification by considering genome-wide IBD (S1 Fig, S1 Table).

In order to fine-map the region on ECA 6 we analyzed the haplotypes in the region of the best associated SNPs in the interval from 80 Mb—84 Mb on ECA 6. We hypothesized that the smallest Shetland ponies were likely to share a haplotype with a size-decreasing derived allele. We identified such a minimal common haplotype of 640 kb in the 19 Shetland ponies with height at withers <87 cm. The flanking SNPs of this haplotype defined a 793 kb critical interval at Chr6:81,004,787–81,798,590 (Fig 1C). This haplotype was shared in homozygous state among all Shetland ponies with height at withers <87 cm and was additionally identified in 16 out of the 29 larger Shetland ponies. Five of these 16 larger Shetland ponies were also homozygous for the haplotype. Their size ranged from 87 to 90 cm.

### Variant identification

In order to identify all genetic variants within this interval we sequenced the genomes of two Shetland ponies, one small (height at withers = 70 cm) and one large (height at withers = 106 cm). The ponies were selected to have opposite genotypes at the 4 top associated SNPs of the GWAS. We called the variants in both Shetland ponies with respect to the EquCab 2 reference assembly. We identified 867 homozygous variants in the small Shetland pony in the critical interval. Only 50 of these were private to the small Shetland pony when we compared them to the variants from the large Shetland pony and 51 other regular-sized horses that were sequenced during the course of other projects. None of the private variants was in the protein-coding region of an annotated gene.

As the critical interval contained the *HMGA2* gene, an excellent functional candidate for the genetic regulation of height, we visually inspected the EquCab reference genome assembly in this region. EquCab2 has a ~1.4 kb gap in the region of the presumed first exon of the *HMGA2* gene. We designed primers in the flanking region of this gap and obtained the missing 1,352 nucleotides of sequence by Sanger sequencing of a PCR product (EMBL accession LN849000). We then inferred the putative full-length coding sequence of the equine *HMGA2* gene from an RNA-seq dataset ([15]; EMBL accession LN849001).

**Fig 2:**
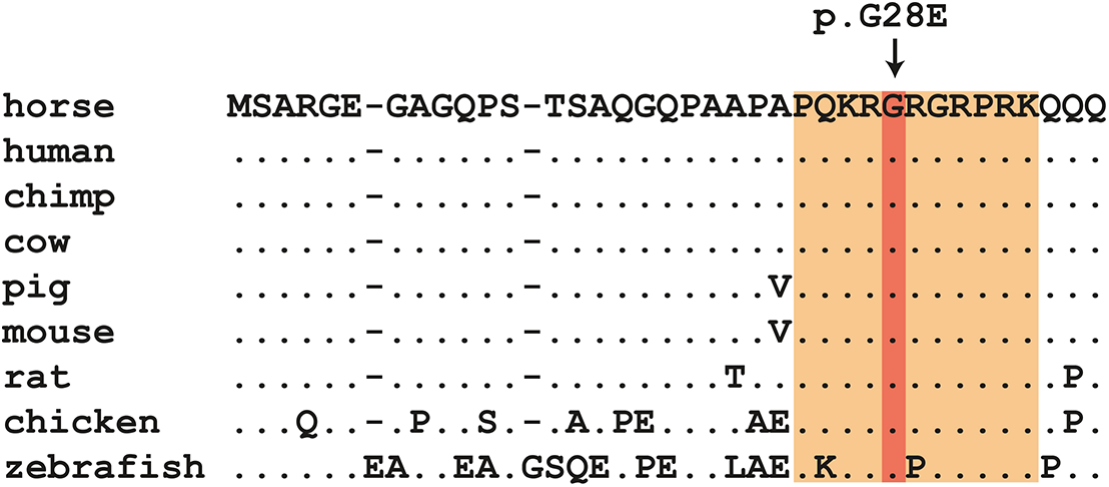
Multiple species alignment of the first 37 amino acids of HMGA2. The first AT-hook domain of HMGA2 is shaded in orange. Position 28 with the p.G28E variant in Shetland ponies is fully conserved in all analyzed vertebrate species including zebrafish The sequences were derived from the following accession numbers: *Equus caballus*, LN849001; *Homo sapiens*, NP_003474.1; *Pan troglodytes*, XP_009423258.1; *Bos taurus*, XP_002687694.2; *Sus scrofa*, XP_005664014.1; *Mus musculus*, NP_034571.1; *Rattus norvegicus*, NP_114459.1; *Gallus gallus*, NP_990332.1; *Danio rerio*, NP_997845.1.

As the NGS data did not contain any variant calls in the gap region, we PCR-amplified a region containing the first exon from the two Shetland ponies used in the initial variant analysis. We sequenced the PCR products using the Sanger method and identified an additional non-synonymous variant, *HMGA2*:c.83G>A. This variant is predicted to result in the exchange of a neutral glycine residue to a negatively charged glutamate in the HMGA2 protein (p.G28E). The major HMGA2 protein isoform is highly conserved across vertebrates and contains 109 amino acids. It is a DNA-binding protein with 3 so-called AT-hooks, strongly positively charged domains, which bind to the minor groove of DNA. The p.G28E exchange is located in the first of these AT-hooks. We hypothesized that the additional negative charge would impair the binding of this domain to the likewise negatively charged DNA. PolyPhen-2 also predicted this variant to be probably damaging (score = 0.999) [16]. The extremely high conservation of the first AT-hook of HMGA2 is shown in Fig 2.

### Validation of the association

**Fig 3:**
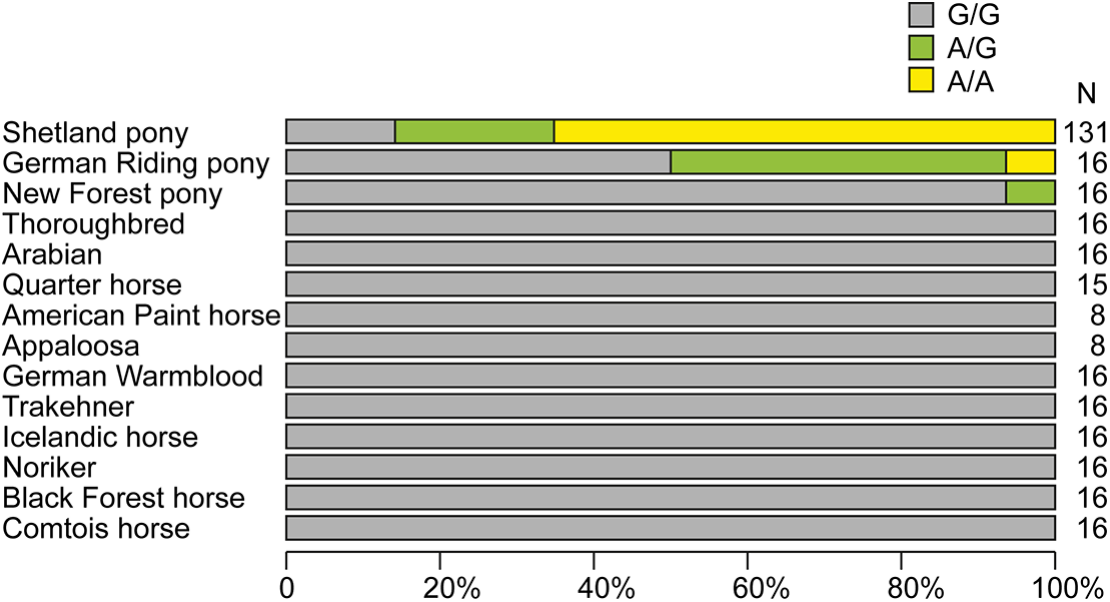
A: Proportion of each genotype for the *HMGA2*:c.83G>A variant by breed. The mutant allele occurred exclusively in small pony breeds.

We re-sequenced the *HMGA2*:c.83G>A variant in a larger cohort of 131 Shetland ponies (including the 48 animals from the initial GWAS) and in 223 animals of 11 other horse and 2 other pony breeds. The analysis confirmed that the mutant allele occurs exclusively in Shetland and the two other pony breeds, but not in full-sized horses (Fig 3).

We then added the *HMGA2*:c.83G>A genotypes to our SNP chip data and repeated the GWAS in the 48 Shetland ponies used for the initial QTL detection. In the new analysis *HMGA2*:c.83G>A was the best-associated marker in the genome. When we corrected for the effect of this marker by including its genotype as a co-variable in a mixed-model analysis, the entire association signal on ECA 6 disappeared, indicating that this variant alone is responsible for the detected height QTL (S1 Fig).

### Genotype-phenotype correlation

**Fig 4:**
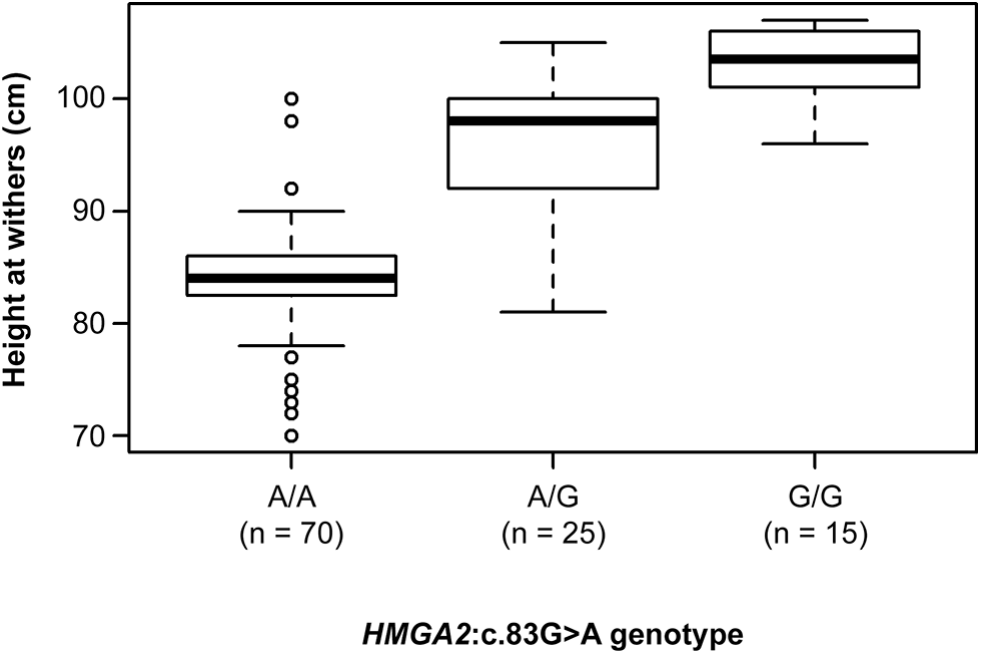
Genotype-phenotype correlation. The box plot displays the height at withers of Shetland ponies with the three different genotypes at the *HMGA2*:c.83G>A variant. The medians, which are indicated as solid horizontal lines in the box plot, were A/A: 84 cm, A/G: 98 cm, G/G: 104 cm. The mean heights per genotype were: A/A: 84 cm, A/G: 95 cm, G/G: 103 cm.

We obtained height measurements on 110 genotyped Shetland ponies. The average height at withers of these animals was 89.3 cm. We then compared the height distributions between the different genotype classes at *HMGA2*:c.83G>A. The differences between all three genotype classes were significant (p < 2.5 x 10^−4^). The correlation plot suggested a largely additive mode of inheritance with a mean reduction of the height at withers of 9.5 cm per copy of mutant A allele (Fig 4).

### Functional analysis of the protein

**Fig 5:**
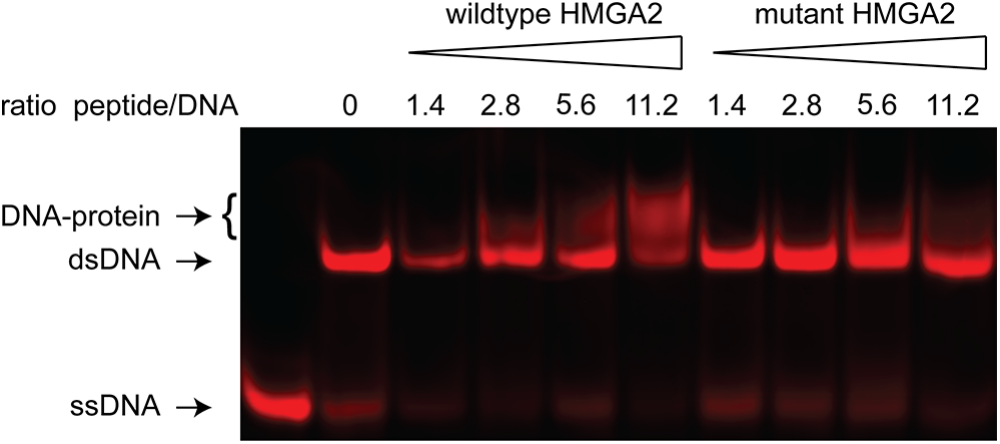
Electrophoretic mobility shift assay (EMSA). We incubated a double-stranded oligonucleotide (dsDNA) with increasing concentrations of peptides containing either the wildtype (^28^Gly) or mutant (^28^Glu) sequence of the first AT-hook of the *HMGA2* protein. With the wildtype sequence a partial shift of the dsDNA band could already be observed at the lowest concentration of peptide corresponding to a molar ratio of peptide to DNA of 1.4. At the highest concentration of the wildtype peptide, the majority of the DNA was in the DNA-protein complex. In contrast, with the mutant peptide the band shift was only observed at higher peptide concentrations and a smaller proportion of DNA was bound.

To assess the functional consequences of the amino acid exchange we performed an electrophoretic mobility shift assay (EMSA). We incubated peptides comprising 11 amino acids with the first AT-hook domain of either the wildtype or mutant HMGA2 protein with a double-stranded oligonucleotide containing an HMGA2 binding site. The EMSA experiment showed that the mutant peptide bound with greatly reduced affinity to the DNA compared to the peptide with the wildtype sequence (Fig 5).

## Discussion

We performed a genome-wide association study for height based on 48 Shetland ponies and 41,683 SNPs. The SNPs were genotyped on the Illumina equine SNP70 (“70 k”) chip. This genotyping array covers the end of ECA 6, which is known to be lacking on the 50 k SNP chip [7]. We were able to detect a height QTL comprising the *HMGA2* locus in this small sample set. The study highlights advantages and disadvantages of working in the non-model species horse.

On one hand horse breeds have an advantageous population structure with limited heterogeneity. Human selection in horses and other domestic animals has enriched interesting alleles with strong phenotypic effects that are much easier to detect than functional variants influencing typical complex traits in humans. This is especially true for body size in the studied population: Shetland ponies probably evolved for more than one thousand years in comparative isolation on the Shetland Islands. Thus, it seems likely that the *HMGA2*:c.83G>A variant arose during this time and was favored by selection in an environment with scarce feed. Later their small body size enabled them to e.g. work in coal mines [17]. Today Shetland ponies with their small size are valued as companion animals and have a global distribution. There is an actively ongoing selection for diverse sizes in Shetland ponies maintaining the frequency of the mutant *HMGA2* allele at an intermediate frequency and providing a unique opportunity to detect functional variants with major effects on body size in this population.

On the other hand the tools and resources for the horse are not yet optimal. The gap in the region of the first exon of the *HMGA2* gene in the current horse genome assembly greatly complicated the identification of the *HMGA2*:c83G>A variant. It is well known that the EquCab 2 horse genome sequence, which was exclusively obtained by Sanger whole genome shotgun sequencing, has many gaps at the GC-rich first exons of genes due to the low cloning efficiency of such regions. Thus this study clearly emphasizes the importance of the currently ongoing efforts to close such gaps by novel sequencing technologies that no longer rely on bacterial cloning.

The *HMGA2* locus has a well-known role in height determination in horses and many other species including humans and dogs. Furthermore, microdeletions in humans, which include *HMGA2* lead to proportionate short growth, whereas overexpression is associated with tumorigenesis [18,19]. *Hmga2* deficient mice display the “pygmy” phenotype and *Hmga1 / Hmga2* double knock-out mice the “superpygmy” phenotype, which is characterized by even smaller body size, but also increased embryonic mortality and reduced lifespan [20,21]. The HMGA2 protein is a DNA-binding protein with a function in transcription regulation [22]. The protein contains 3 AT-hook DNA binding motifs. The AT-hook contains a central RGR motif, which directly contacts the minor groove of DNA [23]. The binding motif is usually PRGRP [24] but in the first AT-hook it is changed to GRGRP. The entire AT-hook contains additional positively charged amino acids, which help to bind the negatively charged DNA. Its optimal DNA binding sequence has been determined in a SELEX study [25]. The equine p.G28E variant identified in our study affects the central glycine of the first AT-hook. It is thus not surprising that this amino exchange drastically reduced its binding affinity in the EMSA. The functional knowledge on HMGA2 from other species and our own functional data strongly support the causality of the *HMGA2*:c83G>A variant for the observed size reduction.

To the best of our knowledge we report the first spontaneous point mutation within the *HMGA2* gene that leads to a pronounced reduction in overall size and thus represents a quantitative trait nucleotide (QTN) for the economically important trait “height at withers” in horses. Our results are in line with previous observations that the enormous size variation observed in many domestic animal species is to a large extent caused by few alleles with very large effects.

## Material and Methods

### Ethics statement

Animal work consisted of height measurements and collecting blood or hair samples of privately owned horses with owners’ consent. The collection of blood samples was conducted in accordance with the relevant local guidelines (Swiss law on animal protection and welfare–permit no. SO 01/12, issued by the veterinary service of the canton of Solothurn). Blood samples were collected by state approved veterinarians.

### Animals, phenotypes, and genotypes

We collected blood and hair samples from 131 Shetland ponies for DNA isolation and measured the height at withers from 110 animals. The height at withers ranged from 70 cm up to 107 cm. We compared the means of the different genotype 3 groups at the *HMGA2*:c.83G>A variant using the wilcox.test in R [26]. We also checked for differences in size regarding sex, however no significant difference (p = 0.289) could be found and thus males and females were analyzed together. DNA from 48 Shetland ponies was genotyped on Illumina equine SNP70 beadchips (GeneSeek/Neogen).

### Genome-wide association study

Genome-wide association studies (GWAS) were performed using plink and using the function mmscore in the GenABEL R package [27, 28]. After filtering for maf (> 0.05), missing genotypes (< 0.1) and deviation of Hardy-Weinberg equilibrium (p > 10^−5^), 41,683 SNPs remained in our data set. We set the significance threshold to 0.05 and applied a Bonferroni correction for multiple testing. The additional genome-wide association study including the *HMGA2*:c.83G>A genotype as covariate in a mixed model was only performed by using the mmscore function in GenABEL [28]. The three R commands for this last analysis were: (1) gcov<-as.numeric(gtdata(data)[,"Ex1_var"]) to define the genotypes at the additional SNP as covariate, (2) h2a.flaxen1 <- polygenic_hglm(Stm~gcov, data.clean, kin = data.clean.gkin) to define the mixed model, and (3) an.mm.AFF1 <- mmscore(h2a.flaxen1, data.clean) to calculate the association.

### Haplotype analysis

We inferred the trait associated haplotype by phasing the genotype data between 80 Mb and 84 Mb on ECA6 with the Shapeit software [29]. Haplotypes were then visually analyzed in order to identify a shared haplotype in the Mini-Shetland ponies.

### Next-generation sequencing

Using Illumina’s TruSeq DNA PCR-free library preparation kit and following the manufacturer’s instructions we prepared libraries with 350 bp insert size from one small (SPH020) and one large Shetland pony (SPH041) and collected one lane of Illumina HiSeq2500 paired-end reads (2 x 100 bp) or roughly 25x coverage per horse. We mapped the reads to the EquCab 2 reference genome using the Burrows-Wheeler Aligner (BWA) version 0.7.5a [30] with default settings and obtained 618,449,856 and 563,994,599 uniquely mapping reads in SPH020 and SPH041 respectively. After sorting the mapped reads by the coordinates of the sequence, we labeled the PCR duplicates also with Picard tools (http://sourceforge.net/projects/picard/). We used the Genome Analysis Tool Kit (GATK version v2.7.4, [31]) to perform local realignment and to produce a cleaned BAM file. Variant calls were then made with the unified genotyper module of GATK (GATK version v2.7.4). Variant data for each sample were obtained in variant call format (version 4.1) as raw calls for all samples and sites flagged using the variant filtration module of GATK. Variant calls that failed to pass the following filters were labeled accordingly in the call set: (i) Hard to Validate MQ0 ≥ 4 & ((MQ0 / (1.0 * DP)) > 0.1); (ii) strand bias (low Quality scores) QUAL < 30.0 || (Quality by depth) QD < 5.0 || (homopolymer runs) HRun > 5 || (strand bias) SB > 0.00; (iii) SNP cluster window size 10. The snpEFF software [32] together with the EquCab 2 annotation was used to predict the functional effects of detected variants. The sequence data of the Shetland ponies were deposited in the short read archive of the European Nucleotide Archive (ENA) under accession PRJEB9267 and PRJEB9269.

### Reference based assembly using Velvet

The gap region in the reference genome (see below) was assembled from the Illumina paired end genome sequence of SPH041. The genome assembler ‘Velvet’ version 1.2.09 [33,34], which includes a novel program called ‘Columbus’ [35] was used for the reference-assisted assembly. BWA version 0.7.5a with default parameters was used for aligning the fastq reads to the Sanger sequenced gap region [30]. These aligned reads where then processed with Velvet Columbus. The k-mer used by Velvet was optimized by test running and eventually set at 37 bp.

### Annotation and gene analysis

The EquCab2 assembly contains a gap in the region of the first exon of the *HMGA2* gene. Consequently, at the time of writing this manuscript the equine *HMGA2* gene was not correctly annotated in any of the major genome browsers (MapViewer, Ensembl, UCSC). We determined a genomic sequence spanning the gap region (EMBL accession LN849000). We then inferred the sequence of the equine *HMGA2* isoform a transcript from equine RNA-seq data [15] and comparisons to the corresponding human RefSeq transcript (NM_003483.4). The sequence of the equine *HMGA2* isoform a transcript was deposited in the EMBL nucleotide database under accession LN849001 and all numbering in our manuscript refers to this accession.

### Sanger sequencing

We used Sanger sequencing to close the gap in the reference genome, to confirm the Illumina sequencing results and to perform targeted genotyping for selected variants. For these experiments we amplified PCR products using AmpliTaqGold360Mastermix (Applied Biosystems). PCR products were directly sequenced on an ABI 3730 capillary sequencer (Applied Biosystems) after treatment with exonuclease I and shrimp alkaline phosphatase. We analyzed the Sanger sequence data with Sequencher 5.1 (GeneCodes). The gap of the horse reference genome containing exon 1 of the *HMGA2* gene spanned about 1.4 kb. We amplified this segment using primers at both ends of the gap with a touchdown PCR protocol (S1 File).

### Electrophoretic mobility shift assay

For the electrophoretic mobility shift assay (EMSA) we used peptides with the sequence of the first AT-hook region. We obtained peptides that were acetylated at the amino-terminus and carried an amide group at the C-terminus from Acris Antibody. The peptide sequences were PQKRGRGRPRK (wildtype) and PQKRERGRPRK (mutant). We ordered an IRD700-labelled oligonucleotide IRD700-CGCTATAAGCGCTAATAACGC from Eurofins genomics and the non-labelled antisense strand GCGTTATTAGCGCTTATAGCG from Microsynth. The sequence for the double-stranded oligonucleotide was derived from a binding site previously described to bind to *HMGA2* [24]. The lyophilized peptides were dissolved in water to a final concentration of approximately 7 μM. The DNA was diluted in water to a concentration of 10 μM and 0.5 μl of labelled primer were annealed with 1.5 μl of unmodified DNA. After annealing the peptide was added in different amounts (1μl, 2μl, 4μl and 8 μl). The volume was adjusted to 10 μl with water and the mixture was incubated for 40 minutes at room temperature (ca. 20°C). Then the reaction was loaded on a 16% acryl amide gel and run for about 1 hour at 80 V. The gel was imaged using an Odyssey® CLx Infrared Imaging System.

## Supporting Information

S1 Fig Manhattan plots from GenABEL using a mixed-model approach. The plot on the left side was calculated including the *HMGA2*:c.83A>G variant. The plot on the right side was calculated using this variant as a covariate in the model.
(TIF)

S1 File PCR primers and conditions. (XLSX)

S1 Table GWAS association data. (XLSX)

## References

[1] Womack JE, Jang HJ, Lee MO. Genomics of complex traits. Ann N Y Acad Sci. 2012;1271: 33–36. 10.1111/j.1749-6632.2012.06733.x 23050961 PMC3483623

[2] Grisart B, Coppieters W, Farnir F, Karim L, Ford C. Positional candidate cloning of a QTL in dairy cattle: identi cation of a missense mutation in the bovine DGAT gene with major effect on milk yield and composition. Genome Res. 2002;12: 222–231. 11827942 10.1101/gr.224202

[3] Locke AE, Kahali B, Berndt SI, Justice AE, Pers TH, Day FR, et al Genetic studies of body mass index yield new insights for obesity biology. Nature. 2015;518: 197–206. 10.1038/nature14177 25673413 PMC4382211

[4] Lango Allen H, Estrada K, Lettre G, Berndt SI, Weedon MN, Rivadeneira F, et al Hundreds of variants clustered in genomic loci and biological pathways affect human height. Nature. 2010;467: 832–838. 10.1038/nature09410 20881960 PMC2955183

[5] Wood AR, Esko T, Yang J, Vedantam S, Pers TH, Gustafsson S, et al Defining the role of common variation in the genomic and biological architecture of adult human height. Nat Genet. 2014; 46: 1173–1186. 10.1038/ng.3097 25282103 PMC4250049

[6] Weedon MN, Lettre G, Freathy RM, Lindgren CM, Voight BF, Perry JRB, et al A common variant of HMGA2 is associated with adult and childhood height in the general population. Nat Genet. 2007;39: 1245–1250. 17767157 10.1038/ng2121 PMC3086278

[7] Makvandi-Nejad S, Hoffman GE, Allen JJ, Chu E, Gu E, Chandler AM, et al Four loci explain 83% of size variation in the horse. Hofreiter M, editor. PLoS One. 2012;7: e39929 10.1371/journal.pone.0039929 22808074 PMC3394777

[8] Rimbault M, Beale HC, Schoenebeck JJ, Hoopes BC, Allen JJ, Kilroy-Glynn P, et al Derived variants at six genes explain nearly half of size reduction in dog breeds. Genome Res. 2013;23: 1985–1995. 10.1101/gr.157339.113 24026177 PMC3847769

[9] Andersson L, Georges M. Domestic-animal genomics: deciphering the genetics of complex traits. Nat Rev Genet. 2004;5: 202–212. 14970822 10.1038/nrg1294

[10] Tetens J, Widmann P, Kühn C, Thaller G. A genome-wide association study indicates LCORL/NCAPG as a candidate locus for withers height in German Warmblood horses. Anim Genet. 2013;44: 467–471. 10.1111/age.12031 23418885

[11] Signer-Hasler H, Flury C, Haase B, Burger D, Simianer H, Leeb T, et al A genome-wide association study reveals loci influencing height and other conformation traits in horses. PLoS One. 2012;7: e37282 10.1371/journal.pone.0037282 22615965 PMC3353922

[12] Weikard R, Altmaier E, Suhre K, Weinberger KM, Hammon HM, Albrecht E, et al Metabolomic profiles indicate distinct physiological pathways affected by two loci with major divergent effect on Bos taurus growth and lipid deposition. Physiol Genomics. 2010;42A: 79–88. 10.1152/physiolgenomics.00120.2010 20647382

[13] Sutter NB, Bustamante CD, Chase K, Gray MM, Zhao K, Zhu L, et al A Single IGF1 Allele Is a Major Determinant of Small Size in Dogs. Science. 2007;316: 112–115. 17412960 10.1126/science.1137045 PMC2789551

[14] Petersen JL, Mickelson JR, Rendahl AK, Valberg SJ, Andersson LS, Axelsson J, et al Genome-Wide Analysis Reveals Selection for Important Traits in Domestic Horse Breeds. PLoS Genet. 2013;9: e1003211 10.1371/journal.pgen.1003211 23349635 PMC3547851

[15] Pacholewska A, Drögemüller M, Klukowska-Rötzler J, Lanz S, Hamza E, Dermitzakis ET, et al The Transcriptome of Equine Peripheral Blood Mononuclear Cells. PLoS One. 2015;10: e0122011 10.1371/journal.pone.0122011 25790166 PMC4366165

[16] Adzhubei IA, Schmidt S, Peshkin L, Ramensky VE, Gerasimova A, Bork P, et al A method and server for predicting damaging missense mutations. Nat Methods. 2010;7: 248–249. 10.1038/nmeth0410-248 20354512 PMC2855889

[17] Bedell FL. The Shetland pony 1st ed. Ames: Iowa State University Press; 1959.

[18] Buysse K, Reardon W, Mehta L, Costa T, Fagerstrom C, Kingsbury DJ, et al The 12q14 microdeletion syndrome: additional patients and further evidence that HMGA2 is an important genetic determinant for human height. Eur J Med Genet. 2009;52: 101–107. 10.1016/j.ejmg.2009.03.001 19298872

[19] Zhang K, Gao H, Wu X, Wang J, Zhou W, Sun G, et al Frequent overexpression of HMGA2 in human atypical teratoid/rhabdoid tumor and its correlation with let-7a3/let-7b miRNA. Clin Cancer Res. 2014;20: 1179–1189. 10.1158/1078-0432.CCR-13-1452 24423609

[20] Zhou X, Benson KF, Ashar HR, Chada K. Mutation responsible for the mouse pygmy phenotype in the developmentally regulated factor HMGI-C. Nature. 1995; 771–774. 7651535 10.1038/376771a0

[21] Federico a., Forzati F, Esposito F, Arra C, Palma G, Barbieri a., et al Hmga1/Hmga2 double knock-out mice display a “superpygmy” phenotype. Biol Open. 2014;3: 372–378. 10.1242/bio.20146759 24728959 PMC4021359

[22] Reeves R. Nuclear functions of the HMG proteins. Biochim Biophys Acta—Gene Regul Mech. 2010;1799: 3–14. 10.1016/j.bbagrm.2009.09.001 PMC2818135 19748605

[23] Huth JR, Bewley C a, Nissen MS, Evans JN, Reeves R, Gronenborn a M, et al The solution structure of an HMG-I(Y)-DNA complex defines a new architectural minor groove binding motif. Nat Struct Biol. 1997;4: 657–665. 9253416 10.1038/nsb0897-657

[24] Fonfría-Subirós E, Acosta-Reyes F, Saperas N, Pous J, Subirana J a., Campos JL. Crystal structure of a complex of DNA with one AT-hook of HMGA1. PLoS One. 2012;7: e37120 10.1371/journal.pone.0037120 22615915 PMC3353895

[25] Cui T, Leng F. Specific recognition of AT-rich DNA sequences by the mammalian high mobility group protein AT-hook 2: A SELEX study. Biochemistry. 2007;46: 13059–13066. 17956125 10.1021/bi701269s

[26] The R Project for Statistical Computing. Available: http://www.R-project.org/.

[27] Purcell S, Neale B, Todd-Brown K, Thomas L, Ferreira M a R, Bender D, et al PLINK: a tool set for whole-genome association and population-based linkage analyses. Am J Hum Genet. 2007;81: 559–575. 17701901 10.1086/519795 PMC1950838

[28] Aulchenko YS, Ripke S, Isaacs A, van Duijn CM. GenABEL: An R library for genome-wide association analysis. Bioinformatics. 2007;23: 1294–1296. 17384015 10.1093/bioinformatics/btm108

[29] Delaneau O, Marchini J, Zagury J-F. A linear complexity phasing method for thousands of genomes. Nat Methods. 2011;9: 179–181. 10.1038/nmeth.1785 22138821

[30] Li H, Durbin R. Fast and accurate short read alignment with Burrows-Wheeler transform. Bioinformatics. 2009;25: 1754–1760. 10.1093/bioinformatics/btp324 19451168 PMC2705234

[31] McKenna A, Hanna M, Banks E, Sivachenko A, Cibulskis K, Kernytsky A, et al The genome analysis toolkit: a MapReduce framework for analyzing next-generation DNA. Genome Res. 2010;20: 1297–1303. 10.1101/gr.107524.110 20644199 PMC2928508

[32] Cingolani P, Platts A, Wang LL, Coon M, Nguyen T, Wang L, et al A program for annotating and predicting the effects of single nucleotide polymorphisms, SnpEff: SNPs in the genome of Drosophila melanogaster strain w1118 ; iso-2; iso-3. Fly. 2012;6: 80–92. 10.4161/fly.19695 22728672 PMC3679285

[33] Zerbino DR, Birney E. Velvet: algorithms for de novo short read assembly using de Bruijn graphs. Genome Res. 2008;18: 821–829. 10.1101/gr.074492.107 18349386 PMC2336801

[34] Zerbino DR, McEwen GK, Margulies EH, Birney E. Pebble and rock band: heuristic resolution of repeats and scaffolding in the velvet short-read de novo assembler. PLoS One. 2009;4: e8407 10.1371/journal.pone.0008407 20027311 PMC2793427

[35] Velvet—Sequence assembler for short reads. Available: http://www.ebi.ac.uk/~zerbino/velvet/.

