# Supplementary figures and images for: A Non-Synonymous HMGA2 Variant Decreases Height in Shetland Ponies and Other Small Horses
Source: PLoS One. 2015 Oct 16;10(10):e0140749. doi: 10.1371/journal.pone.0140749 (PMC4608717; doi:10.1371/journal.pone.0140749)

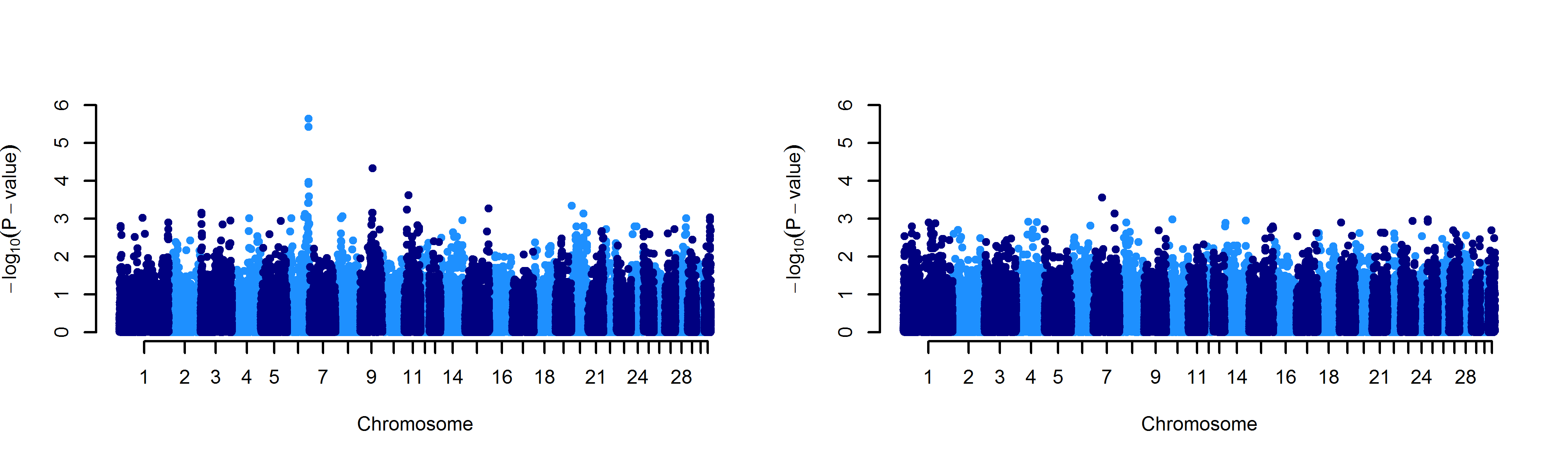

Supplement: S1 Fig — The plot on the left side was calculated including the HMGA2:c.83A>G variant. The plot on the right side was calculated using this variant as a covariate in the model. (TIF) [file pone.0140749.s001.tif]
